# Late presentation of rheumatic heart disease: a justification for renewal of preventive methods?

**Published:** 2009-12-29

**Authors:** Adeseye Abiodun Akintunde, Oladimeji George Opadijo

**Affiliations:** 1 Cardiology Unit, Dept. of Medicine, Ladoke Akintola University of Technology Teaching Hospital, Osogbo Osun State, Nigeria

**Keywords:** Rheumatic Heart Disease, Preventive, Mitral stenosis, Pulmonary Hypertension

## Abstract

Rheumatic heart disease continues to contribute greatly to the burden of cardiovascular disease in Sub-saharan Africa despite advances in medical care. Evidence suggests that the prevalence is far greater than reported. There is therefore the need to emphasize routine screening for at-risk subjects and to appropriately institute adequate therapy and other preventive measures to avert the late and awry manifestation of the disease as presented in this case. This is a case report of one of numerous cases that may eventually suffer the same fate if urgent preventive measures are not instituted. A 20-year old Nigerian woman with past history of recurrent sore throat and a 10-year history of recurrent easy fatiguability and markedly dilated left atrium and pulmonary hypertension presented when she developed severe pulmonary hypertension. A concerted action at all levels of prevention is needed to reduce the burden of this disease among the young population in developing countries.

## Background

Acute rheumatic fever (ARF) is a disease characterized by a systemic inflammatory response that occurs approximately three weeks after an untreated group A beta-haemolytic streptococcal pharyngeal infection. Though the incidence of ARF in the developed world is on the decline, the same is not true of developing nations like Nigeria. Appropriate antibiotic therapy during pharyngeal infection essentially eliminates the future risk of developing rheumatic fever [1].

Diagnosis of ARF is clinical, using the revised Jones criteria of 1992 [2]. Evidence of two major criteria or one major criterion and at least two minor criteria is enough to make a diagnosis. The major criteria include pancarditis, polyarthritis, chorea, erythema marginatum and subcutaneous nodules. The minor criteria include athralgia, fever, elevated erythrocyte sedimentation rate (ESR), increased C-reactive protein and first degree atrio-ventricular block [1]. The most feared consequence of the pathologic processes is chronic scarring leading to valvular stenosis and regurgitations. Treatment may involve the use of aspirin and/or corticosteroids. Prevention of recurrence of carditis and ARF involves long-term administration of benzathine penicillin.

## Case report

O.T, a 20-year old Nigerian female student was seen in July 2007 with history of recurrent breathlessness which had started about 10 years earlier. Her symptoms worsened in the previous three weeks when she became breathless at rest, had paroxysmal nocturnal dyspnoea, bilateral leg swelling and cough productive of whitish, and blood-stained sputum. Past history was significant for recurrent sore throat in childhood and skin rashes. She was not previously diagnosed hypertensive or asthmatic. She was the second child in a monogamous family; both parents are in a very low income group. Her genotype was AS and she was not smoking cigarettes or consuming alcohol. There was no family history of the disease in any of her siblings.

Examination revealed that the woman looked pale, was chronically ill, and had a tinge of jaundice with severe bilateral pitting pedal oedema. A cardiovascular system examination revealed a pulse rate of 120 per minute, irregular and small volume, blood pressure of 90/60mmHg, elevated jugular venous pressure, displaced apex beat which was located at 7th intercostal space anterior axillary line and a fourth, first and second heart sounds. The respiratory rate was 36 cycles per minute and bilateral crepitations. Other examination findings were a tender, pulsatile hepatomegaly of 6 cm below the right coastal margin.

**Figure 1:**
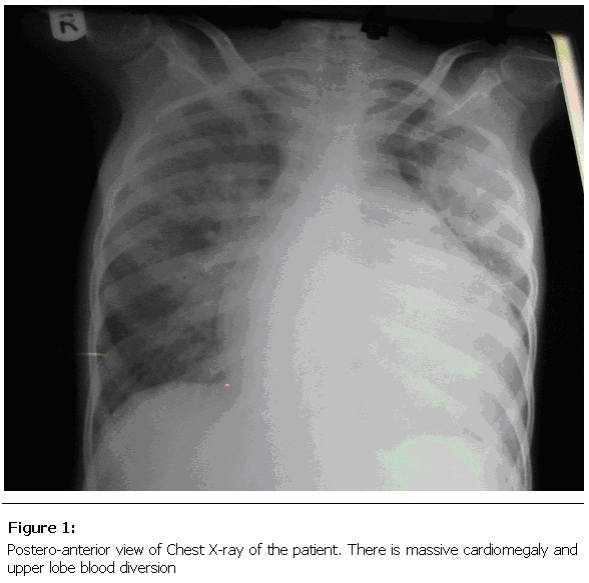
Postero-anterior view of Chest X-ray of the patient. There is massive cardiomegaly and upper lobe blood diversion.

An echocardiography showed densely thickened mitral valves with severe commissural fusion leading to doming of the mitral valve in diastole, markedly dilated left atrium with an intramural clot attached to the posterior left atrial wall, reduced left ventricular ejection fraction, markedly dilated right atrium and right ventricle. Colour flow showed severe tricuspid regurgitation. The calculated mitral valve area is 0.67cm square. Complete blood count showed leucocytosis with a white cell count of 19,700/cubic centimetres and marked neutrophilia. The chest X-ray revealed cardiomegaly with a double cardiac shadow in keeping with the dilated left and right atrium ( Figure 1 ).

She was placed on diuretics (frusemide and low-dose spironolactone) Angiotensin Converting Enzyme inhibitor (Lisinopril), intranasal oxygen, digoxin, subcutaneous clexane and antibiotics. On the fifth day of admission, she suddenly deteriorated and became restless and breathless at rest, at which time the pulse and the blood pressure were not recordable. She thereafter died. The relatives did not consent to autopsy.

## Discussion

Rheumatic fever, no doubt remains a disease with great morbidity and mortality in most low and middle income countries despite having been almost eradicated in high income countries [1]. High frequency and severity of rheumatic heart disease is still been reported in many parts of Africa [2,3].

Rheumatic fever (rheumatic heart disease) is likely to have developed as a non-suppurative complication of group A- beta haemolytic streptococcal pharyngitis due to delayed immune response [4], as it is found in the case presented here. An estimated 1% of all school children in developing countries shows signs of rheumatic heart disease [5].

Epidemiological association between group A beta haemolytic streptococcal throat infection and the subsequent development of acute rheumatic fever in Nigerians have been well established [6]. Sani et al [7], showed that rheumatic heart disease is still a major cause of morbidity among Nigerians and that many already had complications at presentation. There have also been concerns about the possibilities of the non-group A beta haemolytic streptococci to cause rheumatic fever and acute glomerulonephritis [4,5,8].

The case being presented typifies the cost of late presentation in patients with rheumatic heart disease. The cause of death in the case was likely to have been pulmonary thromboembolism due to the intracardiac thrombus and/or deep venous thrombosis seen on echocardiography and the sudden nature of the mortality. Effective screening and treatment at the early stages are more likely to reduce the burden of the disease. Management of rheumatic heart disease involves a great deal of manpower, skills, facilities and financial resources which are very limted in Nigeria [9].

Therefore, prevention at all levels should be aggressively undertaken to reduce to the barest minimum if not eradicate the morbidities and mortalities associated with rheumatic fever/rheumatic heart diseases in Nigeria and other countries in Africa [9].

The Drakenberg declaration on the control of rheumatic fever and rheumatic heart disease in Africa was released from the First Pan African conference of Rheumatic fever and rheumatic heart disease held in South Africa in 2005. It highlighted programmes which include effort to increase awareness of rheumatic fever and rheumatic heart disease among general public and practitioners. This includes the establishment of surveillance programmes to measure the burden of disease in the population, advocacy to increase allocation of resources for the treatment of affected children and young adults, and the implementation of primary and secondary prevention schemes in all countries of Africa [4,10].

Similar reports have emanated from other centres highlighting the burden of rheumatic heart disease [2,9]. Many of the patients come to the hospital when surgery is not a feasible option as in this case with severe pulmonary hypertension. Others that are fortunate to be detected much earlier had to wait for support from philanthropists and organizations for financial support to embark on the overseas journey for the surgical repair. Many die from various complications such as cardiac arrhythmias, thromboembolism, while waiting. Tackling poverty in developing countries therefore remains paramount in eradicating this disease if the millennium development goals must be achieved.

## Conclusion

Rheumatic heart disease remains a disease that is still ravaging the developing economy of the world and paradoxically where resources are scarce to control it. Therefore, prevention and appropriate treatment of rheumatic fever and all febrile illness in childhood is very crucial to eradicating this disease.
